# Mixed Production of Filamentous Fungal Spores for Preventing Soil-Transmitted Helminth Zoonoses: A Preliminary Analysis

**DOI:** 10.1155/2013/567876

**Published:** 2013-04-21

**Authors:** M. S. Arias, C. F. Cazapal-Monteiro, J. Suárez, S. Miguélez, I. Francisco, F. L. Arroyo, J. L. Suárez, A. Paz-Silva, R. Sánchez-Andrade, P. Mendoza de Gives

**Affiliations:** ^1^Equine Diseases Study Group (COPAR, GI-2120), Animal Pathology Department, Veterinary Faculty, Santiago de Compostela University, 27002 Lugo, Spain; ^2^Área de Helmintología, Centro Nacional de Investigación Disciplinaria en Parasitología Veterinaria, Instituto Nacional de Investigaciones Forestales, Agrícolas y Pecuarias, Paseo Cuaunahuac 8534, 62550 Jiutepec, MOR, Mexico

## Abstract

Helminth zoonoses are parasitic infections shared by humans and animals, being the soil-transmitted helminths (STHs) mainly caused by roundworms (ascarids) and hookworms. This study was aimed to assess the individual and/or mixed production of two helminth-antagonistic fungi, one ovicide (*Mucor circinelloides*) and other predator (*Duddingtonia flagrans*). Fungi were grown both in Petri plates and in a submerged culture (composed by water, NaCl, Na_2_HPO_4_ *·* 12 H_2_O, and wheat (*Triticum aestivum*)). A Fasciola hepatica recombinant protein (FhrAPS) was incorporated to the cultures to improve fungal production. All the cultured plates showed fungal growth, without difference in the development of the fungi when grown alone or mixed. High counts of *Mucor* spores were produced in liquid media cultures, and no significant differences were achieved regarding single or mixed cultures, or the incorporation of the FhrAPS. A significantly higher production of *Duddingtonia* spores after the incorporation of the FhrAPS was observed. When analyzing the parasiticide efficacy of the fungal mixture, viability of *T. canis* eggs reduced to 51%, and the numbers of third stage cyathostomin larvae reduced to 4%. It is concluded, the capability of a fungal mixture containing an ovicide (*Mucor*) and a predator species (*Duddingtonia*) for growing together in a submerged medium containing the FhrAPS offers a very interesting tool for preventing STHs.

## 1. Introduction

Helminth zoonoses are parasitic infections that can be shared by animals and humans. Animals become infected after the ingestion of different parasite forms (eggs, cysts, or larvae) passed in the feces by other infected animals. A very important source of disease in humans comes from foodstuffs of animal origin, or through the intimate contact with pets. Nevertheless, transmission of zoonoses can also occur directly from environment by ingestion of infective stages of pathogens with water or food, or even through a direct contact with nature, which points out the need for some action against them. For these reasons, control of infected animals is the main procedure in preventing both animal and people infection [1].

Some of the most common zoonoses are caused by parasites as roundworms (ascarids) and hookworms, also known soil-transmitted helminths [2]. *Toxocara* spp. and *Ascaris* spp. are ascarid nematodes affecting pets (dogs and cats) and pigs (*Ascaris suum*). The adult females release nonembryonated eggs which pass to the soil in the feces. After 2–5 weeks, the second stage larvae (L2) develop inside the egg, which turns infective for the definitive and the paratenic hosts (rodents, humans) [3]. People can become infected when eggs containing viable L2 are ingested [4]. 

Hookworms (mainly *Ancylostoma spp.* and *Uncinaria spp.*) are blood-sucking worm parasites of the small intestine, and occasionally of the colon of dogs and cats [5]. Most human infections result in cutaneous larval migrans or *creeping eruption* caused by third stage larvae in the soil [6, 7]. As the larvae migrate in the skin, a very narrow and red (erythematous) line is visible on the surface of the skin. Many cases originate on the beach of warm climates, such as the coastal areas of the southeast United States and South America [8].

Most frequently measures for the control of zoonoses consist of deworming of pets by providing them commercially available parasiticides [9, 10]. Nevertheless, this seems not enough due to that the presence of eggs and/or larvae in the soil becomes a risk for the human infection, and thus other procedures focused to their elimination seem needed.

In the last two decades, biological measures for controlling some parasitic infections by means of soil fungi have been proposed [11]. The possibility of the simultaneous presence of different parasitic infective stages in the soil should indicate the necessity of several fungal biocontrol agents. Although the antagonism between some fungal species has been pointed [12], the concurrent production of two predator species has been recently reported [13].

While some filamentous fungi are able for breaking the eggshells from certain helminths [14, 15], others are able for capturing and destroying infective larvae [16, 17]. These are organisms innocuous for animals and plants which spread as spores, and large amounts for reaching successful results in the control of parasites look necessary [18].

Two biotechnological processes can be applied for fungal sporogenesis, submerged fermentation, or solid state growth [19–21]. The solid state fermentation (SSF) consists of the fungal growth in a moist solid substrate (natural or inert) in the presence of a little amount of water [22], whereas submerged culture involves their propagation in a liquid medium. Different attempts have been made to select low-cost substrate for spore production in SSF, as Coffee husk, cassava bagasse, or defatted soybean cake [23]. The possible utilization of agroindustrial residues as refuse potato has been also checked [18].

In the current investigation, the main goal consisted of analyzing of the possibility for the combined production of spores belonging to two soil filamentous fungi able to develop activity against helminth eggs (*Mucor circinelloides*) or larvae (*Duddingtonia flagrans*). Then the stimulant effect of addition of a recombinant surface protein from the liver trematode *Fasciola hepatica* (as a nitrogen source) on the spore production has been assessed.

## 2. Materials and Methods

### 2.1. Experimental Design

In the first experiment, fungal species showing different parasiticide activity, *Mucor circinelloides* (ovicide) and *Duddingtonia flagrans* (predator), were jointly grown both in a solid state (in Petri plates) and in a submerged (liquid) culture for discarding they were not antagonists and could develop together.

For trying to enhance the spore production in the submerged medium, a *Fasciola hepatica* surface recombinant protein (FhrAPS) (GenBank database accession AY676331) [24] was added to the liquid culture as a nitrogen source. This medium was called COPFr.

The second experiment consisted of the evaluation of the parasiticide effect of the fungal mixture. Feces of puppies passing eggs of the roundworm *Toxocara canis* were used. Stools were also collected from horses shedding eggs of cyathostomins. As occurs in the hookworms, cyathostomins eggs are passed in the feces to the soil, where they develop until the infective stage, the third stage larvae (L3).

### 2.2. Culture of Fungi in Petri Plates

Fungal spores were produced, harvested, and managed in Petri dishes (8.5 cm diameter) containing corn meal agar (CMA) [25]. The medium was prepared with 20 g agar, 20 g corn wheat flour, and 1 L distilled water. Then, it was autoclaved (121°C, 20 min.), and when the temperature dropped to 37°C, poured into sterile Petri plates.

Agar blocks 7 × 7 mm cut from the colony margin of actively growing stock cultures were used to inoculate experimental cultures. Only one agar block was used for inoculating each plate.

A total of 18 plates were divided into 3 groups of 6 plates/each: one was cultured with *Mucor circinelloides*, another with *Duddingtonia flagrans*, and the third with *Mucor* and *Duddingtonia*. Fungal growth was assessed under a light microscope, by measuring trap and chlamydospore formation per cm^2^ (data not shown).

### 2.3. Production of Fungal Spores in Submerged Culture (COPFr)

The two fungal species were cultured in a liquid medium composed by (per L water) 7.1 g NaCl, 1.6 g Na_2_HPO_4_ · 12 H_2_O, and 30.6 g wheat (*Triticum aestivum*). Once prepared, the medium was sterilized (121°C, 20 min) and kept into 1 L Nalgene bottles, once cooled. For the inoculation of the bottles, fungi spores were scraped from actively growing stock cultures in Petri plates and added to the liquid culture. A total of 8 nalgene bottles with 250 mL liquid medium were utilized, 4 for each of the fungi, control (monospecific), mixed (including both species), FhrAPS (each of the fungi specimens cultured in the presence of the recombinant protein), and mixed-FhrAPS (with the two fungal species).

The approximate counts of spores incorporated to each bottle were 24000/mL belonging to *Mucor*, by 2000/mL to *Duddingtonia*. On the basis of a prior investigation [26], 0.423 mg of FhrAPS (*Fasciola hepatica* surface recombinant protein) was added per L of medium. Production of the recombinant protein was done according to previous results [24].

The numbers of spores per mL were calculated by means of a Neubauer chamber cell counting and a light microscope. In this case, 8 aliquots of 1 mL were collected from each bottle every 7 days.

### 2.4. Measurement of Fungal Sporogenesis

The variations in the spore production in the different groups were determined by calculating two indexes. The Index of Sporogenesis Increment (ISI) was estimated for assessing, in each sampling day, the production of spores in the different cultures in respect to the control one. The Cumulative Index of Sporogenesis Increment (CISI) was calculated for evaluating the linear rise of spore production in each sampling day regarding the beginning of the study (day 0):

(1) $$\begin{array}{c} \text{ISI}\;\;\left(\%\right)=\left[1-\frac{\text{Spore}{\text{s}}_{\text{culture}}}{\text{Spore}{\text{s}}_{\text{control}}}\right]\times 100,\\ \text{CISI}\;\;\left(\%\right)=\left[1-\frac{\text{Spore}{\text{s}}_{\text{culture}}}{\text{Spore}{\text{s}}_{\text{culture}\;\;\text{day}\;0}}\right]\times 100. \end{array}$$

### 2.5. Parasiticide Activity of the Fungal Mixture

The effect of the fungal mixture was evaluated against *Toxocara canis* eggs and cyathostomin third stage larvae. For this purpose, 5 grams of canine feces with a quantity of 1900 *T. canis* eggs per gram of feces (EPG) was placed in plastic boxes and maintained during 30 days under field conditions. Five grams of sand was also added to each box for simulating the conditions of a public park or enjoying area. The treated group consisted of 20 boxes provided 3 mL of COPr containing 3.2 × 10^5^ spores of both *M. circinelloides* and 3.2 × 10^5^ *D. flagrans*; the number of control boxes (without fungi) was 10.

Thirty plastic boxes with five grams of feces of horses passing 600 cyathostomins EPG were utilized for assaying the predator effect of the fungal combination. After maintaining the boxes at 20°C until the presence of third stage larval stages were observed (16 days), twenty (treated group) were added 3 mL of COPr containing 3.2 × 10^5^ spores of both *Mucor* and 3.2 × 10^5^ *Duddingtonia*, whereas 10 remained without fungi as controls.

In each assay, the successful was determined by calculating the percentage of reduction:

(2) $$\begin{array}{c} \%\;\;\text{Reduction}=\left[1-\frac{\text{Parasitic}\;\;\text{stage}{\text{s}}_{\text{day}\;0}}{\text{Parasitic}\;\;\text{stage}{\text{s}}_{\text{day}\;30}}\right]\times 100. \end{array}$$

### 2.6. Statistical Analysis

Data analysis was performed using SPSS 18.0 (SPSS Inc, Chicago, Illinois). Obtained results for the spore counts were tested by one-way analysis of variance (ANOVA) at a significance level of *P* < 0.05. The significant differences were analyzed with Tukey test to find out which group differed from each other.

The values of the indexes of sporogenesis increment (ISI, CISI) were analyzed by the nonparametric Kruskal-Wallis test, and then the *U* Mann-Whitney probe was applied for assessing the significantly different groups.

## 3. Results

### 3.1. Mixed Production of Fungi (Mucor + Duddingtonia)

**Figure 1:**
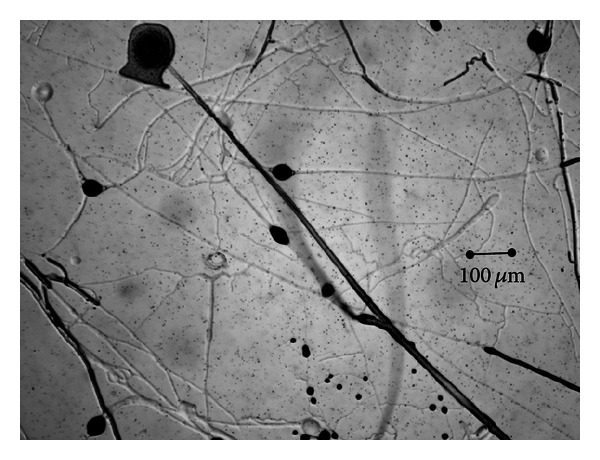
Mixed growth of *Mucor circinelloides* and *Duddingtonia flagrans* in a solid-state medium (Petri plates).

**Figure 2:**
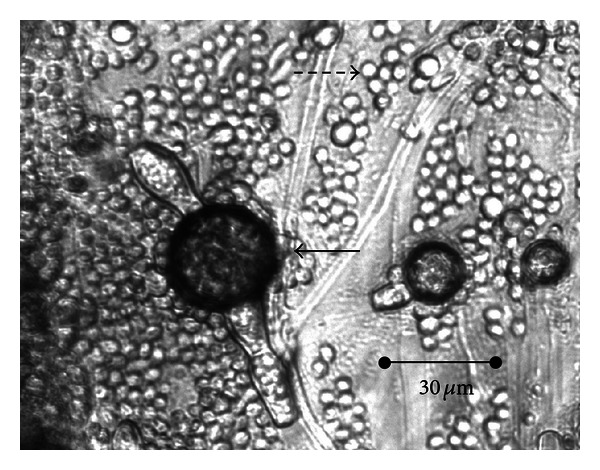
Simultaneous production of spores of *M. circinelloides* (⇢) and *D. flagrans* (→) in solid-state medium (Petri plates).

Fungal growth was observed in all the cultures, and no differences in the development of the fungi when grown alone or mixed were noted (Figures 1 and 2). Production of spores began 14 days after the culture of each of the specimens.

**Figure 3:**
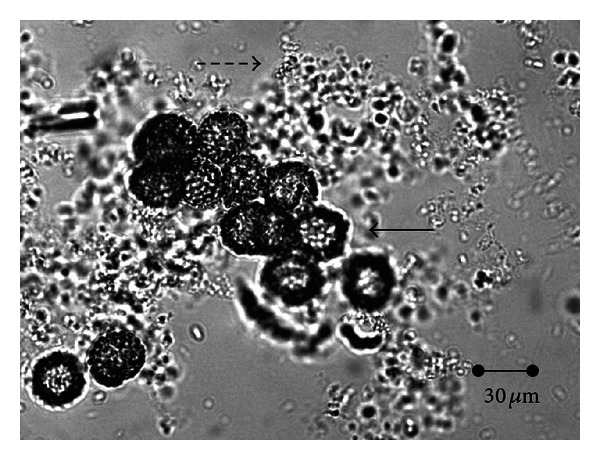
Simultaneous production of spores of *M. circinelloides* (⇢) and *D. flagrans* (→) in submerged (liquid) culture.

**Figure 4:**
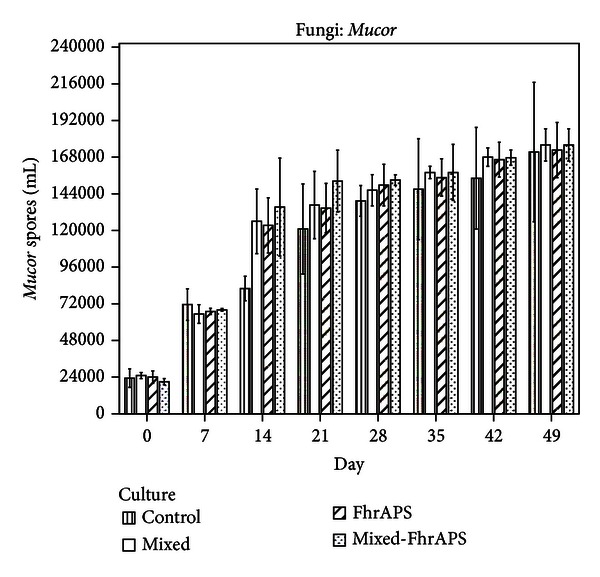
Production of spores of *M. circinelloides* in a submerged culture in the presence of *D. flagrans*.

Analysis of liquid media cultured with *Mucor circinelloides* demonstrated elevated counts of spores (Figures 3 and 4). Similar numbers in all the groups were recorded and no significant differences achieved regarding single or mixed cultures, or the incorporation of the FhrAPS.

The highest increase in the sporogenesis was recorded between 7 and 14 culture days, as demonstrated by the ISI values (Table 1), and counts higher than 120,000 spores/mL were recorded. Significant differences in the ISI values between the mixed and the mixed-FhrAPS cultures were obtained, as well as between the FhrAPS and the mixed-FhrAPS media.

Production of spores became slow to the end of study, when counts above 168,000 spores/mL were achieved. The CISI values were higher than 80% from the 21st day (Table 1). The differences in the CISI percentages between the mixed and the mixed-FhrAPS cultures and between the FhrAPS and the mixed-FhrAPS media were significant (*U* = −2.108, *P* = 0.035 and *U* = −2.047, *P* = 0.041, resp.).

**Figure 5:**
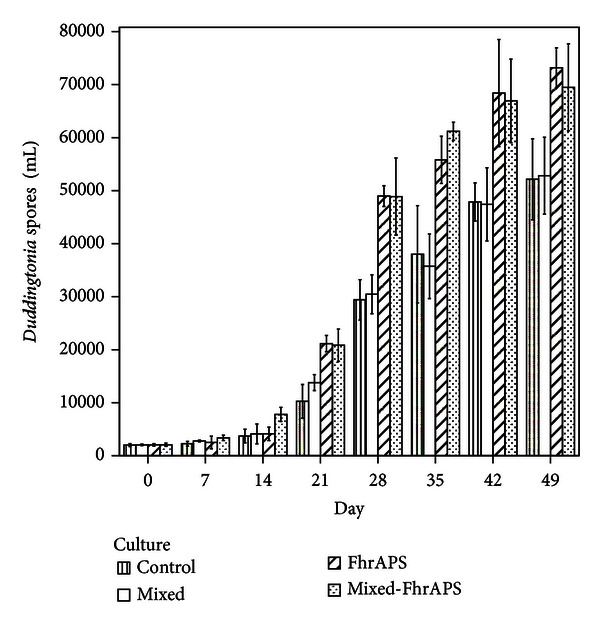
Production of spores of *D. flagrans *in a submerged culture in the presence of *M. circinelloides*.

Figure 5 summarizes the changes in the production of *Duddingtonia* spores. Similar numbers in the four groups until the 14th day were recorded, and significantly highest quantities in the cultures containing the two fungal species and the FhrAPS were demonstrated (9000 spores/mL) (*F* = 9.934, *P* = 0.001).

A noteworthy increment in the spore formation in all the groups by the 21 days of culturing was recorded, especially for that containing the FhrAPS (Table 1), in which numbers near to 50000 spores/mL were obtained (*F* = 22.546, *P* = 0.001).

After the 28th day of study, sporogenesis rose significantly in the cultures incorporating the FhrAPS recombinant protein, and the highest numbers were obtained at the 49th day (>65,000 spores/mL). 

The ISI percentages ranged between 30 and 52% for the FhrAPS group and between 25 and 51% for the mixed-FhrAPS (Table 1). Significant differences between the ISI values in mixed and FhrAPS and between mixed and mixed-FhrAPS were demonstrated (*U* = −2.505, *P* = 0.012 and *U* = −3.006, *P* = 0.003, resp.).

The CISI reached values ≥ 80% in all the cultures from the 21st day, and no differences were obtained (Table 1).

*Post hoc* Tukey analysis indicated the incorporation of the FhrAPS to the culture medium increased significantly the production of *Duddingtonia* spores, whereas no effect on *Mucor* was recorded (Table 2).

### 3.2. Parasiticide Effect of the Fungal Combination (Mucor + Duddingtonia)

As summarized in Table 3, in the boxes containing *T. canis* eggs and provided the fungal mixture, viability of the eggs reduced to 51%. Significant differences regarding the controls were observed (*P* < 0.05).

The percentage of reduction of third stage cyathostomin larvae in the boxes added the fungal mixture was 96%, and significant differences with the controls were demonstrated (Table 3).

## 4. Discussion

Control of soil-transmitted helminth zoonoses (STHs) should merge the deworming of animals together with some action against the parasitic infective stages in the soil. On considering that the main STHs are caused by ingestion of eggs containing infective stages (roundworms) or through the skin penetration of third stage larvae (hookworms), a combined activity should be required. In the current research, the possibilities of the biological control based on the combination of parasiticide fungi were analyzed. Firstly, two specimens developing activity against different infective stages, *Mucor* (ovicide) and *Duddingtonia* (predator), were jointly cultured in Petri plates and then by submerged fermentation. No signs of antagonism between these fungi were observed, and a very extensive growth for both *Mucor* and *Duddingtonia* was achieved. These results disagree with that reported for other soil specimens showing antagonistic effect [27, 28].

Within a period of 3–6 weeks to several months, *T. canis *eggs develop to the infectious stage that can survive for at least one year under optimal circumstances [29]. Development of *Ancylostoma* eggs to L3 takes several weeks regarding the environmental conditions (temperature, humidity) [30]. In the present investigation, the parasiticide activity of the fungal mixture against *T. canis* was assessed. Third stage cyathostomin larvae were also used due to that these are parasites developing an identical external phase to the hookworms in their life cycle. The viability of *T. canis* eggs reduced to 50% in the presence of the fungal mixture, and 96% reduction in cyathostomin L3 have been obtained thirty days after the addition of a mixture of *Mucor* + *Duddingtonia* spores, in agreement with previous investigations [26]. The ability of a *Duddingtonia flagrans* isolate for destroying L3 of *Ancylostoma spp*. in about 48 hours has been previously reported, whereas the addition of *Mucor hiemalis* to *T. canis* eggs did not exhibit ovicidal activity [13, 31].

Several seem the points needing consideration for the practical utilization of fungi in the biological control of parasitic stages in the soil, most importantly appropriate distribution, and large-scale production of spores [23]. There is scarce information available on the application of fungal spores to the soil. Spraying has been considered very interesting for large-scale procedures, but the loss of spores through “bounce-off” and run-off effects and the nonhomogeneous spread of the spray reduce its accuracy [32]. Production of spores in submerged cultures seems more appropriate for being distributed by spraying, watering, or irrigation methods, providing very useful tools for the dissemination of spores on sandy areas (beaches) and/or recreation facilities.

Only few data are available regarding spores production, but better yield, morphology, and high stability by means of solid state fermentation have been pointed [33]. In the current work, elevated counts of spores belonging to the two fungal biocontrol agents in the submerged culture were obtained. After spreading *Duddingtonia* spores (2 · 10^6^/100 m^2^) directly onto the soil in a paddock, a significant prevention on the challenge of horses was observed, as demonstrated by a 78%–90% reduction in the counts of cyathostomin eggs [34]. Taking into account that 1 L yielded ca. 70 · 10^6^ in the submerged culture, this volume should provide an adequate amount for about 35 m^2^.

Methods for commercial production of spores are usually done on organic solid substrates (cereal grains, rice) or inert supports (starch-based substrates as agar). In this last case, a liquid nutritive media must soak the inert substrates [35]. A great alternative to achieve a satisfying price is the utilization of industrial residues or agricultural products, and different nutrient types in the agar media resulted in variability in the number of spores produced [36].

Fungi utilized in the current research are applied to soil as spores, and the relationship between sporogenesis and the quantity and nature of carbon and nitrogen sources available in a culture media has been previously stated [37]. There has been reported a beneficial effect of nitrogen on the mycelial growth, necessary for optimum sporulation [38]. In the present investigation, a *Fasciola hepatica* surface recombinant protein (FhrAPS) was added to the submerged culture, and significantly higher counts of spores of both fungal specimens were yielded, in coincidence with data obtained after soaking Petri plates with antigens from different helminth parasites [25]. In this case, the FhrAPS comprises a small protein (25 aac) very easy to obtain by using bacteria growth media when needed and in the precise amount.

As stated before, prevention of STHs involves several features, as deworming of pets following veterinary prescription [39], better education of pet owners to avoid the presence of their feces in public areas, and then rapid removal. Some measures have been suggested with the aim to limit the possibilities for the development of *T. canis* or *Ancylostoma* eggs until reaching the infective stage. While frequent replacement of sand or fence construction could be appropriate, these measures are not easily applied, and thus prevention would be deficient [40]. 

Ground areas from public parks in urban or suburban areas can be highly contaminated by *T. canis* eggs or *Ancylostoma* larvae, probably because almost all the ground is paved; thus pets frequent the soil zones as gardens and/or sandpits [30, 41, 42]. Other possible measure for preventing human infection could be dissemination of fungal spores when watering or fertilizing the plants. It should be considered that both *Mucor* and *Duddingtonia* are not hazardous for animal or vegetal specimens, but they are effective against vegetal parasites also.

## 5. Conclusions

The capability of a fungal mixture containing an ovicide (*Mucor*) and a predator species (*Duddingtonia*) for growing together offers a very interesting tool for preventing STHs by destroying the parasitic infective stages in the soil. Culturing the fungi in a submerged medium in the presence of a recombinant protein from the trematode *Fasciola hepatica* (FhrAPS) gives enough spores to be applied in the soil by dipping or watering. 

## Figures and Tables

**Table 1:** Variations in the spore production in the different groups. ISI (Index of Sporogenesis Increment): production of spores, in each sampling day, with respect to the control one. CISI (Cumulative Index of Sporogenesis Increment): linear rise of spore production, in each sampling day, regarding the beginning of the study (day 0). Index values are expressed as percentages.

Index	Day	*Mucor circinelloides *				*Duddingtonia flagrans *			
		Control	Mixed	FhrAPS	Mixed-FhrAPS	Control	Mixed	FhrAPS	Mixed-FhrAPS
ISI (%)	7		0	0	0		19	10	33
	14		35	34	39		10	10	53
	21		11	10	21		26	52	51
	28		5	7	9		3	40	40
	35		7	5	7		0	32	38
	42		8	7	8		0	30	28
	49		3	1	3		1	29	25

	Statistics		*χ* ^2^ = 0.486, *P* = 0.784				*χ* ^2^ = 11.077, *P* = 0.004		

CISI (%)	7	67	62	64	69	11	27	18	39
	14	72	80	81	84	46	50	50	73
	21	81	82	82	86	80	85	90	90
	28	83	83	84	86	93	93	96	96
	35	84	84	84	87	95	94	96	97
	42	85	85	86	87	96	96	97	97
	49	86	86	86	88	96	96	97	97

	Statistics	*χ* ^2^ = 6.298, *P* = 0.098				*χ* ^2^ = 1.982, *P* = 0.593			

**Table 2:** *Post hoc* Tukey analysis of the production of fungal spores.

	*Mucor circinelloides *	*Duddingtonia flagrans *	
Culture	Subset for *α* = 0.05	Subset for *α* = 0.05	
	1	2	1
Control	113704	23204	
Mixed	124042	23643	
FhrAPS	125167		34525
Mixed-FhrAPS	128921		35069

*P*	0.7284	0.9993	0.9986

**Table 3:** Parasiticide effect of the spores of a fungal mixture (*Mucor* + *Duddingtonia*) obtained simultaneously in a submerged culture.

	Day	Control	% Reduction	Mh + Df	% Reduction
Viable *T. canis* eggs	0	1900 ± 203	7 ± 6	1900 ± 203	51 ± 6
	30	1755 ± 197		939 ± 135	

		*F* = 11.505, *P* = 0.001			

Viable L3 cyathostomin	0	376 ± 61	6 ± 3	376 ± 61	96 ± 2
	30	350 ± 52		15 ± 8	

		*F* = 15.002, *P* = 0.001			
